# Violence against women and girls in the Sustainable Development Goals

**DOI:** 10.15171/hpp.2017.01

**Published:** 2016-12-18

**Authors:** Bontha V. Babu, Yadlapalli S. Kusuma

**Affiliations:** ^1^Health Systems Research Division, Indian Council of Medical Research, New Delhi, India; ^2^Centre for Community Medicine, All India Institute of Medical Sciences, New Delhi, India

## Dear Editor,

On 25 September 2015, the 193 member states of the United Nations (UN) unanimously adopted the Sustainable Development Goals (SDGs), a set of 17 goals and 169 targets aiming to transform the world over the next 15 years. These goals are aimed to eliminate poverty, discrimination, abuse and preventable deaths, address environmental destruction, and usher in an era of development for all people, everywhere.^1^ This agenda represents a welcome change to their forerunners, the Millennium Development Goals (MDGs). It was acknowledged that the persistence of violence against women even after 15 years of MDGs has undermined the progress on other MDGs, given its crippling effect on women’s ability to contribute to and benefit from broader developmental processes.^2^ Though, most of the indicators of gender equality have also been improved after implementing MDGs, gender disparities still exist.^2^ More than 30% of women worldwide have experienced either or both physical and sexual violence.^3^ Hence, in SDGs, the prevention of violence against women and girls took an important place, compared to MDGs. Also this enables strong leadership and advocacy that are required for motivation and commitment of financial and other resources.

The SDG-5 exclusively deals with achieving gender equality and empowering women and girls. Though it’s the legacy of MDG-3, this SDG targeted to eliminate all forms of harmful practices and violence against women and girls and appropriate targets are set (Table 1). The SDG-16, which is meant for promoting peaceful and inclusive societies for sustainable development, included a goal to end abuse, exploitation, trafficking and all other forms of violence against and torture of children (Table 1). This goal targets reducing violence and related deaths among women covering deaths related to domestic violence and dowry, which are rampant in many developing countries.^4^ These two SDGs through four targets (5.2, 5.3, 16.1 and 16.2) directly addressed violence against women and girls, and there are several targets among the other SDGs that were aimed directly or indirectly to prevent and reduce violence against women and girls (Table 1).

**Table 1:** Sustainable Development Goals and targets related to prevention and elimination of violence against women and girls

**SDG/Target**	**Description**
SDG 5	Achieving gender equality and empowering women and girls
Target 5.2	Eliminate all forms of violence against women and girls including trafficking and sexual and other forms of exploitation
Target 5.3	Eliminating all harmful practices such as child, early and forced marriages and female genital mutilation
SDG 16	Promoting peaceful and inclusive societies for sustainable development
Target 16.2	Ending abuse, exploitation, trafficking and all forms of violence against and torture of children
Target 16.1	Reducing all forms of violence and related deaths to attain a peaceful and inclusive societies
**Goals and targets influence preventing violence indirectly**	
Target 1.4	Ensuring that all men and women have equal rights to resources, basic services, ownership and control over properties, inheritance, natural resources, technology, etc.
Target 1b	Creating policy frameworks based on gender-sensitive developmental strategies
Target 2.3	Doubling agricultural productivity/income in small-scale food producers, particularly women through secure and equal access to land and other resources
Target 3.7	Ensuring universal access to sexual and reproductive health care and related services
Target 4.1	Ensuring that all girls and boys complete free, equitable and quality primary and secondary education
Target 4.2	Ensuring that all children have access to quality early childhood development, care and pre-primary education
Target 4.3	Ensuring equal access to affordable quality technical, vocational and tertiary/university education
Target 4.5	Eliminating gender disparity and ensuring equal access in education
Target 5.1	Ending all forms of discrimination against women and girls everywhere
Target 5.4	Recognising and valuing unpaid care and domestic work carried out by women
Target 5.5	Ensuring women’s participation/leadership in decision making in all spheres
Target 5.6	Ensuring universal access to sexual and reproductive health and reproductive rights
SDG 5a	Undertaking reforms to give women equal rights and access to economic resources
SDG 5b	Enhancing technology to empower women
SDG 5c	Adopting and strengthening policies and enforceable legislation towards gender equality and women empowerment
Target 11.2	Providing universal access to safe, affordable, accessible and sustainable public transport
Target 11.7	Providing universal access to safe, inclusive and accessible public spaces, particularly for women and children

Thus, SDGs gave enough importance and called for the reduction of “all forms of violence everywhere.” In addition, it identified different social and political enablers of gender equality that eventually reduce violence against women and girls. These enablers are addressed under different SDGs (Table 1). Thus, SDGs recognised the impact of violence against women and girls on well-being of women in specific and on development agenda in general,^5^ and acknowledge tacitly that violence against women and girls is preventable. This is an important recognition to the campaigns against gender-based violence. It is known that violence and other abusive behaviours against women and girls are widespread and are rooted in the gender inequalities of power and resources. And these inequalities were institutionalised through governmental policies, laws and societal norms that grant preferential rights to men and denial to women.^6^ Hence, it needs comprehensive and multi-faceted efforts to prevent and end this violence. It requires the integration of health promotion and population health strategies. Interventions must be taken at various levels of the society, viz. individuals, families and communities. They must include policy reforms addressing violence directly and indirect drivers like economic and gender inequalities; reorient and prepare the public health system and engaging communities for violence prevention. Multi-sectorial programmes engaging multiple stakeholders seem to be successful to transform deeply entrenched attitudes and behaviours.^7^ Such programmes not only challenge the acceptability of violence, but also address underlying risk factors.^7^ Several opportunities and plans are available to gear up the actions against violence.^6^

The UN General Assembly declared that, all countries and all stakeholders through a revitalised global partnership, mobilise the means required to implement the agenda, focussed in particular on the needs of the poorest and the most vulnerable. This agenda needs to be matched by adequate funding and renewed efforts to mobilise innovation, science and technology.^1^ However, equitable distribution across regions and nations is to be ensured during these campaigns and funds disbursement. Jeffrey Sachs, based on his experience as a special adviser for the MDGs, mentioned four domains in which SDGs should improve upon the attainment of MDGs. They are: inclusion of intermediate objectives and milestones for the 15 years of SDGs, investment in real-time reporting system for SDGs to produce reliable data, engagement of private sector and investing adequately by societies worldwide.^8^ The advantage of inclusion of violence against women and girls in SDGs would be increased intensity of campaigns against violence and more funds for their campaigns and research. Evidences based on existing interventions and programmes show that preventing violence against women and girls is possible. However, implementation of these interventions and programmes require robust information and evidence as the basis. Governments should strengthen routine reporting on violence against women and girls and improve the health systems to assume potential role in responding to violence against women and girls. Accountability to implement these programmes should lie with governments and governments should identify the programme indicators for monitoring and surveillance.^9^ Though all member countries of UN adopted these goals, more than 600 million women live in countries where domestic violence is not recognised as a crime and in 53 countries marital rape is not abusive.^6^ In several countries, the legal systems, customary laws and societal norms foster systematic discrimination against women; and in some countries the health systems are not so matured to tackle the problem of violence with health promotion perspective and the system’s responsiveness is very poor. In such countries and instances, implementing the agenda and achieving the goals is harder. Hence, the real action of designing and implementing interventions at various levels right from global to local communities is the immediate need and subsequently is to scaling-up and intensify these actions to end the violence against women and girls.

## Ethical approval

None to be declared.

## Competing interests

Nothing to declare.

## Authors’ contributions

BVB and YSK jointly conceived this paper, and prepared and revised the manuscript.
